# Antifungal activity of an artificial peptide aptamer SNP-D4 against *Fusarium oxysporum*

**DOI:** 10.7717/peerj.12756

**Published:** 2022-02-22

**Authors:** Junjun Huang, Dan Wang, Hong Li, Yanqiong Tang, Xiang Ma, Hongqian Tang, Min Lin, Zhu Liu

**Affiliations:** 1 College of Life Science Hainan University, Haikou, Hainan, China; 2 Biotechnology Research Institute, Chinese Academy of Agricultural Sciences, Beijing, China

**Keywords:** *Fusarium oxysporum*, Peptide aptamer, Antifungal activity, Aldehyde dehydrogenase, Molecular docking, Protein–protein interactions (PPIs)

## Abstract

*Fusarium oxysporum f. sp. cubense* (FOC4) is a pathogen of banana fusarium wilt, which is a serious problem that has plagued the tropical banana industry for many years. The pathogenic mechanism is complex and unclear, so the prevention and control in agricultural production applications is ineffective. SNP-D4, an artificial peptide aptamer, was identified and specifically inhibited FOC4. To evaluate the efficacy of SNP-D4, FoC4 spores were treated with purified SNP-D4 to calculate the germination and fungicide rates. Damage of FOC4 spores was observed by staining with propidium iodide (PI). Eight proteins of FOC4 were identified to have high affinity for SNP-D4 by a pull-down method combined with Q-Exactive mass spectrometry. Of these eight proteins, A0A5C6SPC6, the aldehyde dehydrogenase of FOC4, was selected as an example to scrutinize the interaction sites with SNP-D4. Molecular docking revealed that Thr66 on the peptide loop of SNP-D4 bound with Tyr437 near the catalytic center of A0A5C6SPC6. Subsequently 42 spore proteins which exhibited associations with the eight proteins were retrieved for protein-protein interaction analysis, demonstrating that SNP-D4 interfered with pathways including ‘translation’, ‘folding, sorting and degradation’, ‘transcription’, ‘signal transduction’ and ‘cell growth and death’, eventually causing the inhibition of growth of FOC4. This study not only investigated the possible pathogenic mechanism of FOC4, but also provided a potential antifungal agent SNP-D4 for use in the control of banana wilt disease.

## Introduction

Banana fusarium wilt is a soil-borne vascular disease caused by *Fusarium oxysporum f. sp*. *cubense* (FOC4). It infects banana roots and causes devastating damage to the banana industry (Ploetz, 2006). The virulence and pathogenicity of FOC4 is far higher than other banana pathogens (Molina et al., 2009). FOC4 has strong tolerance to stress and survives in soil for 40 years (Olivia et al., 2018), causing extensive destruction of fields. FOC4 adheres to root epidermal cells in the form of mycelium, macroconidia, and small conidia, and destroys the membrane structure of roots by secreting a series of toxins. Subsequently FOC4 directly invades plant vascular bundles and induces vascular bundle ligation (De Ascensao & Dubery, 2000). There is still no effective protection against banana fusarium wilt (Guo et al., 2014). The relevant prevention strategies of FOC4 are mainly dependent on the screening of chemical pesticides, however, the excessive dependence and abuse of chemical pesticides brings great harm to the environment and can even endanger human health. So, environmentally friendly alternatives need to be developed for the control of FOC4.

Peptide aptamers (or pepaptamers) are short peptides that bind to target proteins with high specificity and affinity (Hoppe-Seyler et al., 2004). Their structures consist of a stable scaffold protein and a variable peptide loop with both ends immobilized on the scaffold protein (Borghouts, Kunz & Groner, 2008). The variable peptide is usually composed of 8–20 random amino acids, which recognizes and binds to the specific target protein. The development of the artificial pepaptamers are currently used as biological antibacterial agents as they have the following three advantages. First, its target specificity is extremely high so that the pepaptamer distinguishes the different members from the same protein family. Second, its molecular weight is smaller than an antibody, while the binding ability is similar to that of the antibody. Third, it folds into a stable tertiary conformation *in vivo*, which is more conducive to maintain the higher biological activity than general polypeptides.

At present, the pepaptamer has been exploited in tumor therapy (Schilling et al., 2017), food safety (Dong et al., 2014), and plant biotechnology (Sera, 2017). The pepaptamer is widely used for the controls of plant viruses, which hinders the viral cycle in the infected plants. Using geminivirus replication protein (Rep) as the target, a pepaptamer library was screened by yeast two-hybrid analysis, and then the pepaptamer coding sequence was transformed into tomato plants. Transgenic plants were resistant to yellow leaf curl virus and tomato mottle virus (Reyes et al., 2013).

The use of pepaptamers to control plant fungal diseases remains to be explored. Previously we constructed a pepaptamer library using *Staphylococcal* nuclease (SNase, SN) as the scaffold, wherein 16 random amino acids were inserted into the plasmid pTRG (abbreviated as pTRG-SNP). After the plasmids were transformed into *E. coli*, they expressed the fusions of SNase and various peptides acting with diverse structures and functions (abbreviated as SNPs, hereinafter) (Liu et al., 2016). One artificial peptide aptamer, designated as SNP-D4, was tested to interact with calmodulin for inhibiting *Magnaporthe oryzae* (Xu et al., 2019). In this study, SNP-D4 was found capable of specifically impeding the spore germination of FOC4. Afterwards, a total of eight proteins were identified to directly interact with SNP-D4. Of these eight proteins, A0A5C6SPC6, the aldehyde dehydrogenase of FOC4, was chosen as an instance to examine the interaction sites with SNP-D4. Subsequently 42 spore proteins which exhibited high affinities with the eight proteins were retrieved for protein-protein interaction analysis, demonstrating that SNP-D4 interfered in the pathways including ‘translation’, ‘folding, sorting and degradation’, ‘transcription’, ‘signal transduction’ and ‘cell growth and death’, eventually resulting in the inhibition of growth of FOC4.

This study helps to further understand the pathogenic mechanism of FOC4 and contribute to the development of transgenic plants or an antifungal agent against FOC4, bringing a new breakthrough in the control of banana wilt disease.

## Materials and Methods

### Materials

#### Plasmids and strains

The peptide aptamer library pTRG-SNP was constructed (Liu et al., 2016), which expressed both scaffold protein and the random exposed loop of 16 random amino acids. The plasmid pTRG-SN only containing the scaffold was used as the negative control. The vector pET-28a was used to express SNP or SN for antifungal assays.

*Escherichia coli* XL1-Blue MR was used as the host for checking the activity of pTRG-SN expression. *E. coli* BL21(DE3) strain was chosen as the host for pET-28a derivative expression. The pathogen *Fusarium oxysporum f. sp. cubense* tropical race4 (FOC4) strain was stored at 4 °C for use.

#### Medium

LB liquid medium contained 1% tryptone, 0.5% yeast extract, and 0.5% NaCl. LB solid medium was made by supplementing 1.5% agarose into liquid medium. LB medium was adjusted to pH 7.0 and sterilized at 121 °C for 20 min. Potato Dextrose Broth (PDB) medium was purchased from Solarbio (Solarbio Science & Technology Co., Ltd., Beijing, China). PDA medium was appended with 3.7% agar into PDB. PDA and PDB media were adjusted at pH 6.5 and sterilized at 115 °C for 20 min.

### Methods

#### Selection of peptide aptamer for the inhibition of FOC4

The plasmids pTRG-SNP was transformed into *E. coli* XL1-Blue MR, and the clones were picked randomly on the plate and cultured at 37 °C overnight in LB medium supplemented with 5 mM IPTG. The cells were then harvested and suspended in 10 mM PBS (pH 7.4) for ultrasonic disruption. The lysate was mixed with 10 μL of 4 × 10^6^ cells/mL spores of FOC4, and the percent of spore germination was calculated after 6 h incubation at 28 °C. The germination rate was counted using the following formula: Germination rate = (germinated spores/total spores) × 100%. The colonies conferring the inhibition activity to FOC4 were selected and the extracted plasmids were sent for DNA sequencing (Xu et al., 2019).

#### Prokaryotic expression

The functional peptide aptamer SNP-D4 was inserted into pET-28a vector and transformed into *E. coli* BL21 (Liu et al., 2016). The final concentration of 100 μM IPTG (isopropyl β-D-thiogalactoside) was added for induction after the clone was cultured until the optical density (OD) value of 0.4 at 580 nm wavelength. The lysate was loaded onto the nickel column for purification with a gradually increased elution using imidazole (Liu et al., 2016). Likewise, the scaffold protein SN was inserted into pET-28a which enabled His-tagged expression as the negative control.

#### Antifungal assay

The purified SNP-D4 was diluted to the ultimate concentrations of 2–8 μM. Each 10 μL of purified protein and 10 μL of PDB spore suspension with a final concentration of 4 × 10^6^ cells/mL were mixed and incubated at 28 °C for 12 h. Both PBS buffer and SN served as the controls. The spore germination was observed under an optical microscope with a 40× objective lens. Spore germination rates were calculated as described above.

#### Fungicidal assay

The mixtures including both SNP-D4 and 3 × 10^4^ cells/mL spore suspension were cultured in PDB medium at 28 °C for 12 h, followed by plating on PDA medium for an additional 48 h. The number of colonies growing on the plate was observed and the fungicidal rate was calculated as follows: Fungicidal rate % = [(number of negative control colonies – number of treated colonies)/number of negative control colonies] × 100%. Each treatment was repeated three times.

#### PI staining

Both 5 μM SNP-D4 and 2 × 10^6^ cell/mL spores were incubated in the volume of 800 μL PBS at 28 °C for 3 h. The spores were collected by centrifugation at 14,000 rpm for 10 min and resuspended in 200 μL PBS. Prior to washing with 800 μL PBS buffer three times, the spores were incubated with 20 μL PI (100 μg/mL) for 5 min. Thereafter the spores were observed whether they were damaged with a fluorescence microscope equipped with 535 nm excitation and 615 nm emission light sources (Chen, Cruz & Mochly-Rosen, 2015; Crowley et al., 2016).

#### Pull-down assay and mass spectrometry

The purified SNP D4 protein was incubated with unpurified spore lysate to isolate the interacting proteins (Qi et al., 2015). FOC4 was cultured in PDB medium, and the hyphae was filtered by gauze to collect the spores. Subsequently the spores were disrupted with liquid nitrogen and resuspended in 50 mM Tris-HCl (pH 7.0) supplemented with 0.1% Triton X-100. The supernatant was incubated with SNP-D4 for 2 h and loaded on the nickel column. After the nonspecific binding proteins were eluted with 20 mM imidazole, the target proteins interacting with SNP-D4 were pulled down eluted using 80 mM imidazole. Consequently, the elution was performed by Q-Exactive Mass Spectrometry analysis (Thermo Fisher Scientific, Waltham, MA, USA).

#### Molecular docking model of SNP-D4 and spore proteins

To obtain the interactive sites, the three-dimensional model of the protein was created by I-TASSER server (https://zhanglab.ccmb.med.umich.edu/I-TASSER/). Molecular docking was performed using Hex 8.0.0 to evaluate and optimize the docking model. The interactive sites between the ligands and receptors molecules, and the formations of hydrogen bonds were analyzed using the Discovery Studio software.

#### Prediction of protein–protein interactions

The interolog method was used to infer the interactions between spore proteins. *Arabidopsis thaliana*, *Saccharomyces cerevisiae*, *Caenorhabditis elegans*, *Drosophila melanogaster*, *Escherichia coli*, and *Homo sapiens* were selected as reference organisms. Their protein sequences were downloaded from the UniProt (UniProt, 2019) database, and the experimentally verified protein-protein interactions (PPIs) were collected from the BioGrid (Oughtred et al., 2019), IntAct (Orchard et al., 2014), DIP (Salwinski et al., 2004) and MINT (Licata et al., 2012) databases. In addition, *A. thaliana* and *H. sapiens* PPIs were also collected from the TAIR (Berardini et al., 2015) and HPRD (Goel et al., 2012) databases, respectively. Orthologs between FOC4 and the six reference organisms were identified using Inparanoid Version 4.1 (Sonnhammer & Ostlund, 2015), of which the ones with an Inparanoid score < 1.0 were removed. The experimentally verified PPIs of the six reference organisms were identified in FOC4 on the basis of the orthologs, and eventually the interactions between spore proteins were obtained.

### Statistical analysis

Each treatment was performed in triplicate under the same conditions. The experimental data were documented as the mean and standard deviation (mean ± SD). *P* values of less than or equal to 0.05 or 0.01 represented significant or extremely significant differences, respectively.

## Results

### Identification of peptide aptamers for the inhibition of FOC4

**Figure 1:**
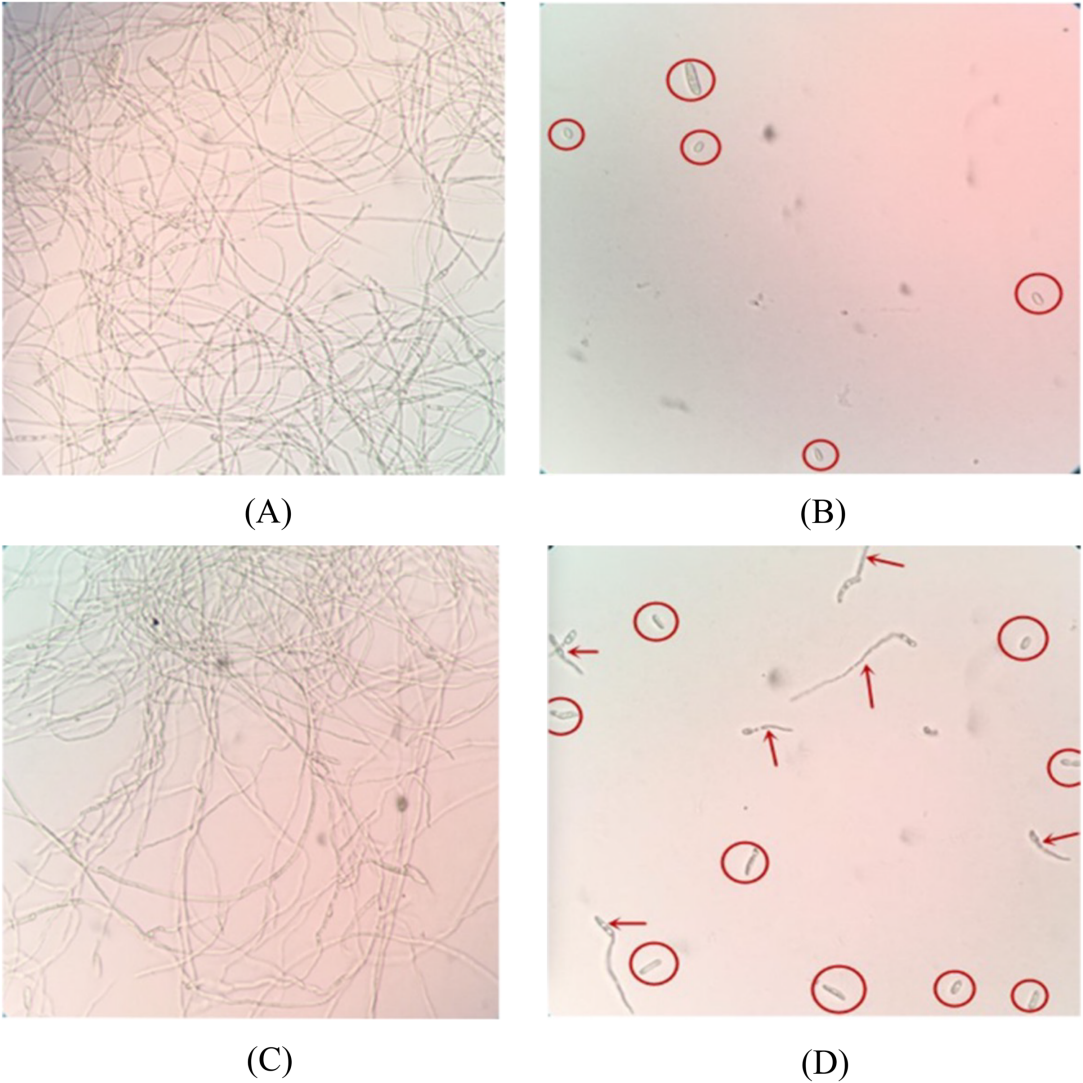
Microscopic analysis of antifungal activity of SNP-D4 against FOC4. The ungerminated spores were marked by red circles, and the germinated spores were marked by red arrows. (A) PBS: Phosphate buffer saline, (B) 0.3% hymexazol, (C) SN: Scaffold protein, (D) SNP-D4: Functional peptide aptamer.

Among 150 peptide aptamer clones, five candidates were found to have inhibitory effects on FOC4 spores, and SNP-D4 had the best inhibitory effect. Both bacterial protein extract of the strain expressing pTRG-SN and PBS treatment promoted significant mycelium growth, indicating that these had no inhibitory effects on the germination of spores. Most of the spores in the SNP-D4 treatment did not germinate, and the hypha length of a few germinated spores were significantly shorter than that of the negative control; The inhibitory effect of SNP-D4 was almost equal to that of 0.3% hymexazol (Fig. 1), indicating that SNP-D4 itself had a certain inhibitory effect on the germination of spores. The sequencing results showed that the peptide aptamer encoded an exposed loop except for the scaffold protein SN, whose amino acid sequence consisted of “VTFLVNTYPNGVQSRA”.

### Antifungal assay of the functional peptide aptamer

**Figure 2:**
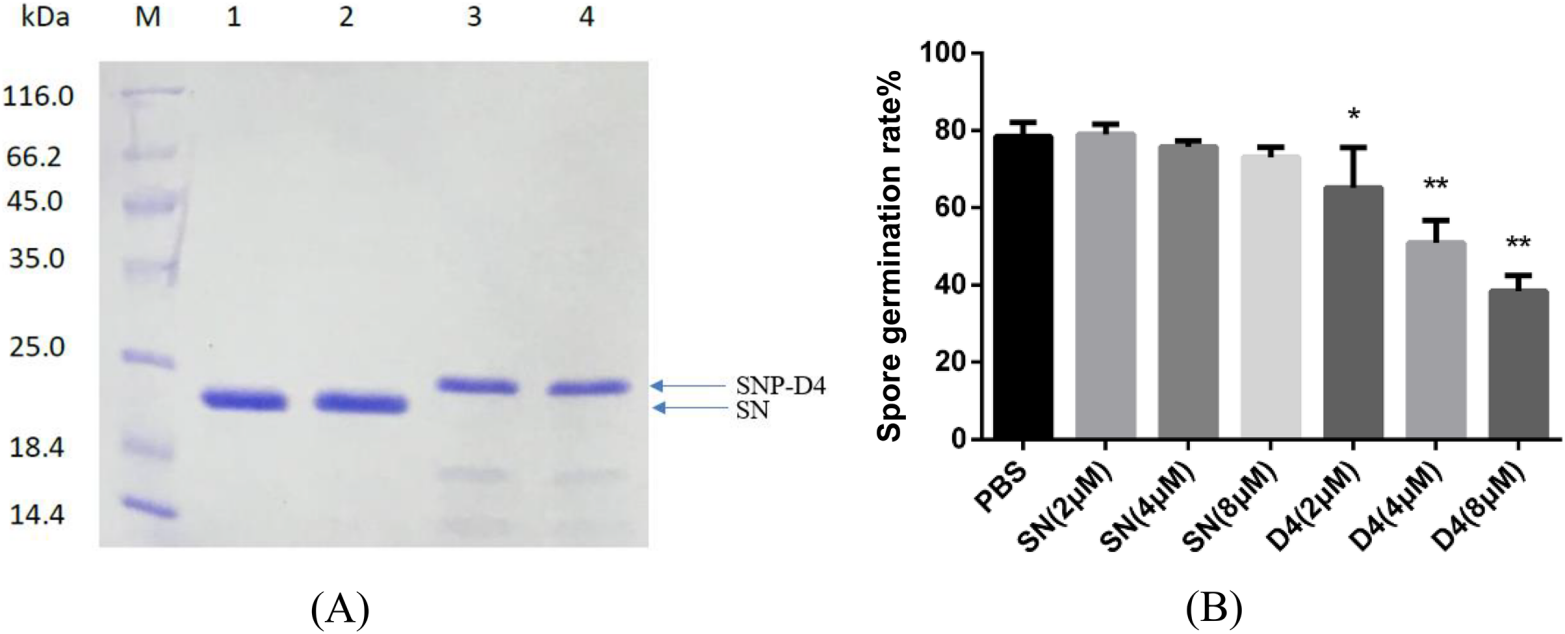
Antifungal assay of the functional peptide aptamer. (A) The purified SNP-D4 and SNP were detected by 12% SDS-PAGE gel. Lane M, molecular mass marker; lanes 1–2, the purified SN; lanes 3–4, the purified SNP. The purification of SN and SNP-D4 were in Fig. S1A and S1B. (B) Effects of SN and SNP-D4 on the spore germination of FOC4. PBS, the treatment with PBS as the negative control; The raw data was in Table S2. An asterisk (*) represented the significant difference at the 0.05 level, **represented the significant difference at the 0.01 level. Mean values of the treated spores were compared to PBS treatment group.

The peptide aptamer SNP-D4 and the scaffold protein SN were purified using nickel column (Fig. 2A). To analyze the inhibition activity on the spores of FOC4, SNP-D4 or SN was diluted to different concentrations and incubated with the spores. The spore germination of FOC4 was significantly inhibited (*p* < 0.05) at 2 μM SNP-D4, and the extremely significant inhibition occurred when elevating the concentration of peptide aptamer to 4 or 8 μM; an antifungal effect was not found by altering SN concentrations (Fig. 2B).

### Fungicidal assay on SNP-D4

**Figure 3:**
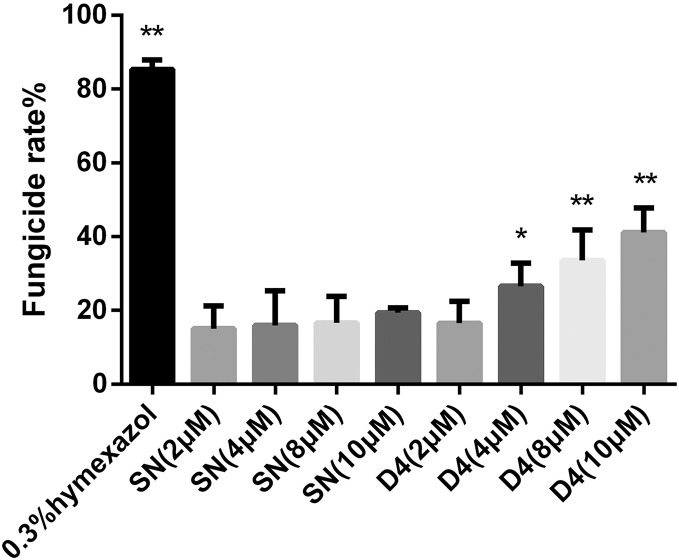
Effects of SN and SNP-D4 on the fungicidal rate of FOC4 spores. The fungicidal rate was measured after different treatments. The raw data was in Table S3. An asterisk (*) represented the sig nificant difference at the 0.05 level, **represented the significant difference at the 0.01 level. Mean values of the treated spores were compared to SN (2 μM) group.

To verify whether SNP-D4 had fungicidal activity, the spores of FOC4 were treated with SNP-D4 and then plated on nutrient-rich PDA medium. The positive control, 0.3% pesticidehymexazol, caused 80% of growth defects on spores, while the scaffold protein SN incurred 20% of the abnormal growth without the concentration dose dependence. SNP-D4 showed fungicidal effects on the spores of FOC4 (Fig. 3), *i.e*., in comparison with the SN groups, the fungicidal rate had a significant difference (*p* < 0.05) at 4 μM SNP-D4, and it reached extremely significant levels (*p* < 0.01) when treated with 8 μM and 10 μM SNP-D4.

### SNP-D4 damages FOC4 spores

**Figure 4:**
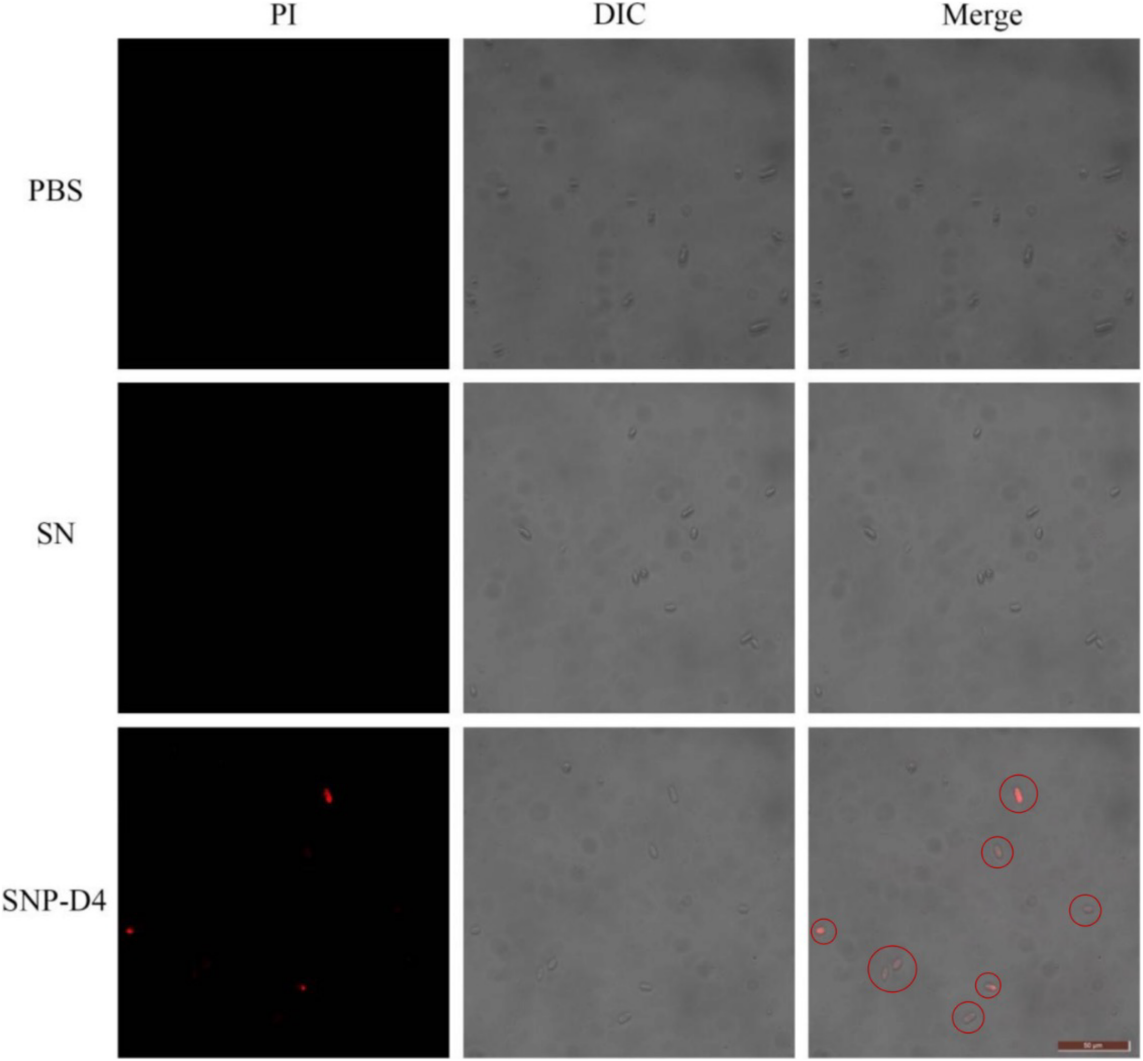
SNP-D4 destroyed FOC4 spores. FOC4 spores were treated with different chemicals, stained with PI, and observed with a fluorescence microscope. The concentrations of SNP-D4 and SN were 10 μM. The damaged spores were marked by red circles. The length of the tuler is 50 μm.

The method of PI staining is used to detect cell death since it enters the interior of the cell and intercalates into double-stranded DNA when the cell membrane is damaged (Shan et al., 2016). The spores were stained with PI dye to determine the survival status of spores. FOC4 spores treated with PBS buffer and SN did not emit red light using fluorescence microscopy, indicating that they had no damage. However, about 50% of the FOC4 spores treated with SNP-D4 (10 μM) emitted red fluorescence, suggesting that the spores were severely damaged and died (Fig. 4).

### Identification of spore proteins interacting with SNP-D4 by mass spectrometry

The collected elution was analyzed by Mass Spectrometry to identify the unique proteins interacting with SNP-D4. A total of eight proteins were identified by searching in the UniProt database. Seven of the proteins were classified with exact functions, while the other one was unknown (Table 1, Fig. S2).

**Table 1:** The proteins interacting with SNP-D4 by mass spectrometry identification.

Accession number	Description	Pathway
A0A559KXS2	DnaJ like subfamily A member 2	Folding, sorting and degradation
A0A5C6TEI9	Rap-GAP domain-containing protein	Unknown
A0A559LNA3	Eukaryotic translation initiation factor 3 subunit K	Unknown
A0A5C6SPC6	Aldehyde dehydrogenase	Metabolism of terpenoids and polyketides; Global and overview maps
A0A5C6SMF1	Sphinganine-1-phosphate aldolas	Signal transduction
A0A5C6SNW8	Unknown	Unknown
A0A5C6TD26	Superkiller protein 8	Folding, sorting and degradation
A0A5C6THD3	Sister chromatid cohesion protein PDS5	Unknown

### Verification of SNP-D4 and the protein A0A5C6SPC6 interaction

**Figure 5:**
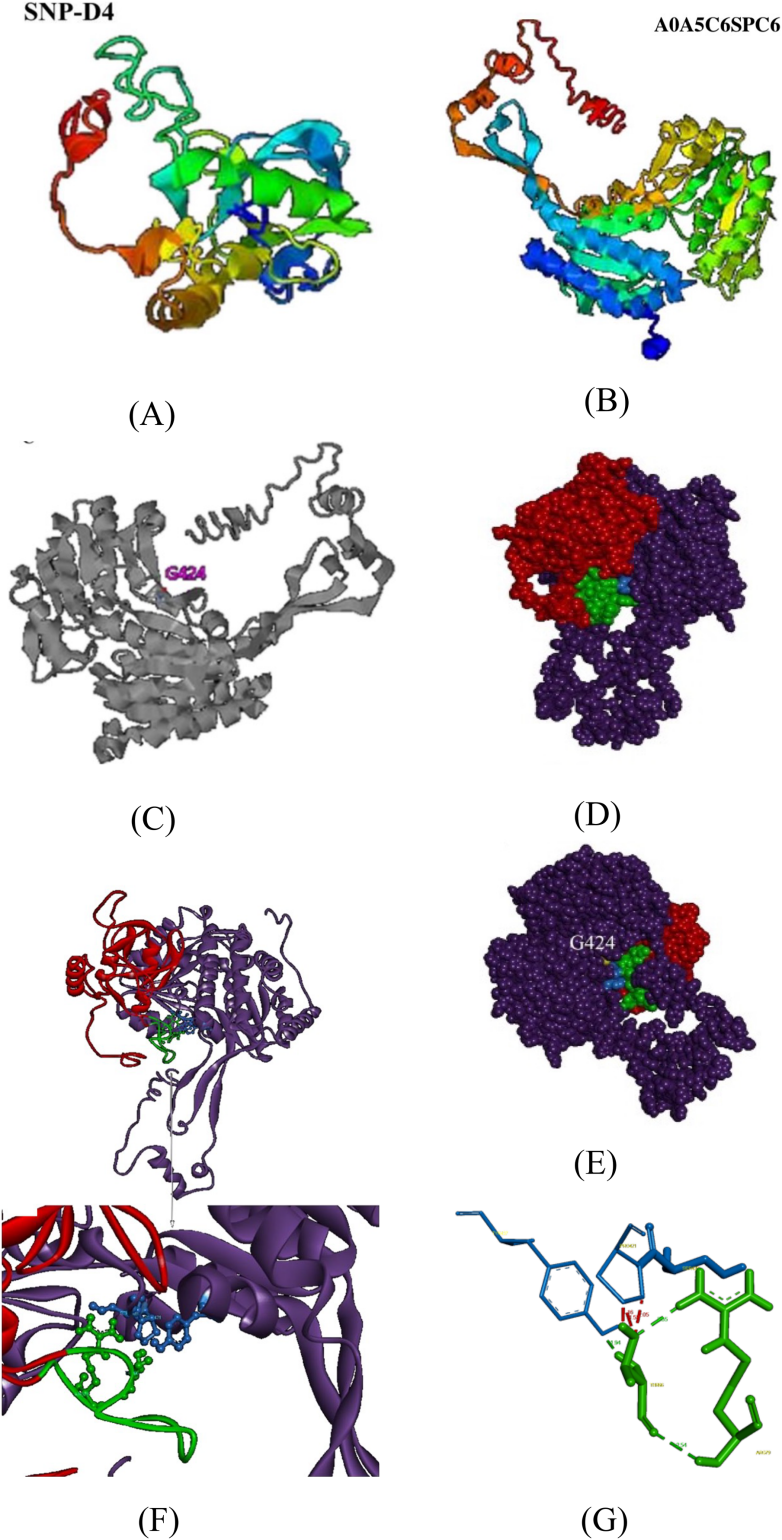
Molecular docking of SNP-D4 and A0A5C6SPC6. (A) and (B) are the protein model of SNP-D4 and A0A5C6SPC6. (C) With G424 as the active center, it formed an enzyme catalytic domain with the surrounding amino acids. (D) Front view and (E) side view of A0A5C6SPC6. (F) The structure of the molecular docking model, and the predicted binding sites. (G) SNP-D4 interacted with Tyr437 (near the catalytic center of A0A5C6SPC6) *via* Thr66 on the peptide loop. The red atoms and structure represented the peptide aptamer scaffold protein of SNP-D4, the green atoms and structure represented the peptide loop of SNP-D4, the purple atoms and structure represented A0A5C6SPC6 protein, the blue atoms and structure represented possible binding sites, the yellow atoms represented the predicted active site.

To further validate the association between SNP-D4 and the protein A0A5C6SPC6, a three-dimensional model of the protein was constructed, and molecular docking was developed. Since the crystals of SNP-D4 and FOC4 A0A5C6SPC6 were not found in the PDB database (Protein data bank, https://www.rcsb.org/), three-dimensional structures were predicted by I-TASSER, yielding the five greatest possibilities of structure. The highest score model of SNP-D4 had C-score of −1.34, Estimated TM-score of 0.55 ± 0.15, and Estimated RMSD of 8.2 ± 4.5 Å (Fig. 5A); the highest score model of FOC4 A0A5C6SPC6 had C-score of −0.10, Estimated TM-score of 0.70 ± 0.12, and Estimated RMSD of 7.6 ± 4.3 Å (Fig. 5B); all the values met the requirements for further structure analysis.

By I-TASSER homology comparison of the crystal structure of A0A5C6SPC6, the enzymatic site of A0A5C6SPC6 was Gly424, which formed a protein active pocket with other amino acids in the region, as shown in Fig. 5C. Molecular docking between SNP-D4 and A0A5C6SPC6 was achieved *via* Hex 8.0.0. E_total_ of the best conformation was −832.66, and the estimated energy was below 0 indicating that the docking result was reliable (Fig. 5E). Through the molecular docking model (Figs. 5F and 5G), the residue Thr66 on the peptide loop of SNP-D4 formed hydrogen bonds with Tyr437 in A0A5C6SPC6 at 1.94 Å. The amino acids Arg79 and Thr66 in SNP-D4 formed two hydrogen bonds with distances of 2.35 Å and 2.54 Å, respectively, stabilizing the structure of the peptide loop and helping SNP-D4 to bind with A0A5C6SPC6. Although there was competition for space between Pro421 and Tyr437 in A0A5C6SPC6, the hydrogen bond between Thr66 in SNP-D4 and Tyr437 in A0A5C6SPC6 was much stronger, and the bonds between Arg79 and Thr66 in SNP-D4 could assist in binding. Besides, the residues Ser420 and Pro421 in the catalytic active center region of A0A5C6SPC6, were close to the crucial site Tyr437 in A0A5C6SPC6. SNP-D4 might contend with Tyr437 at the entrance of the active pocket in A0A5C6SPC6, preventing the substrate from entry. Therefore, SNP-D4 could achieve an antifungal effect.

### Protein–protein interactions effect on the functional pathways in FOC4

**Figure 6:**
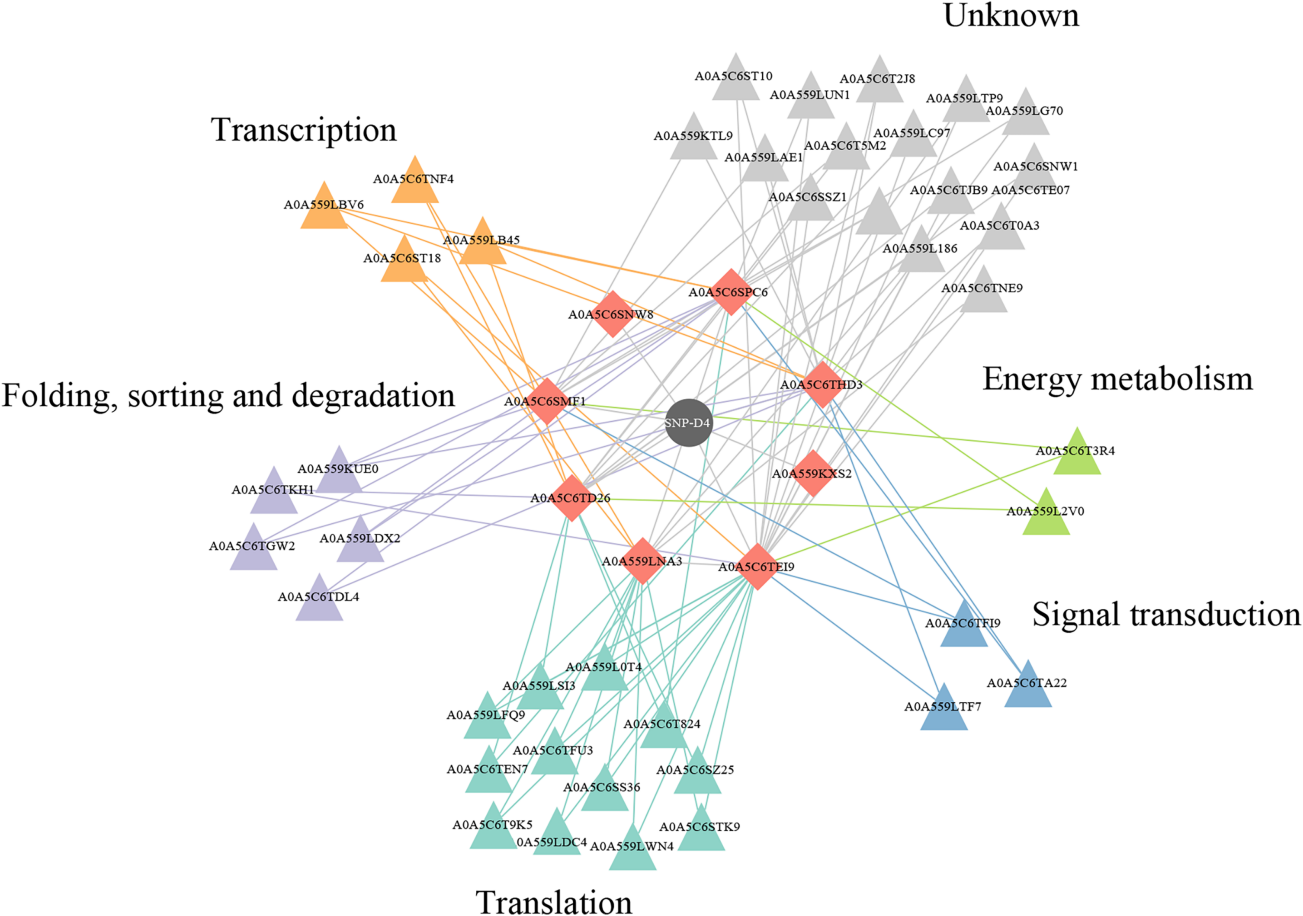
A protein–protein interaction network formed by SNP-D4 and spore proteins. The network was visualized using Cytoscape. In the network, SNP-D4, the proteins interacting with SNP-D4, and the partners were shown as circle, diamonds, and triangles, respectively. The spore protein partners involved in different pathways were displayed in different colors. The interactions between proteins were presented as edges. The raw data were in Table S1.

Two of eight proteins networking with SNP-D4, A0A5C6SNW8 and A0A559KXS2, had no interaction partners in FOC4 based on the interolog method. The remaining six proteins were associated with 42 spore proteins, forming 89 PPIs (Fig. 6, Table S1). The 42 spore proteins were involved in ‘translation’, ‘folding, sorting and degradation’, ‘transcription’, ‘signal transduction’ and ‘cell growth and death’ pathways by KEGG Automatic Annotation Server (https://www.genome.jp/kaas-bin/kaas_main). In the six proteins networking with SNP-D4, A0A559LNA3, A0A5C6SPC6, A0A5C6TEI9 were associated with each of the five pathways. While for A0A5C6THD3, A0A5C6SMF1, A0A5C6TD26, they were only connected to four of the five pathways.

## Discussion

Banana wilt disease is one of the important fungal diseases that endangers the banana industry and has long attracted people’s attention (Ploetz, 2006). With the rapid mutation of pathogenic fungi, traditional control methods have been challenged. To effectively prevent the blight that is harmful to the banana industry, it is still necessary to explore new and effective environmental protection techniques.

Peptide aptamers, as a new type of biomaterial, are increasingly being applied in drug development and as antiviral agents in biological pesticides (Lopez-Ochoa, Ramirez-Prado & Hanley-Bowdoin, 2006). The peptide aptamers are screened for the purpose of interfering with virus function such as the assembly of viral DNA replication complexes (Reyes et al., 2013). Previously, the peptide aptamer SNP-D4 was verified to target calmodulin for the growth inhibition of *Magnaporthe oryzae* (Xu et al., 2019). In this study, SNP-D4 was identified to specifically repress the pathogen of *Fusarium oxysporum* f. sp. *cubense*. To evaluate the antifungal activity, SNP-D4 was expressed in *E. coli*, purified, and the inhibitory effect on *F. oxysporum* was performed, revealing that the spore germination of *F. oxysporum* was dependent on the concentration of SNP-D4 (Fig. 2B). Furthermore, the functional region of the peptide aptamer was the variable peptide segment, not the scaffold protein (Figs. 5A and 5B). A fungicide assay was performed, which determined that SNP-D4 effectively killed pathogenic spores (Fig. 3). Besides, PI staining experiment revealed that the outer membrane of *F. oxysporum* spores was damaged after treating with SNP-D4 (Fig. 4).

To figure out the action of SNP-D4 on FOC4, a pull down assay was executed to capture the target proteins in spores using the purified SNP-D4 with a histidine tag as the prey. The proteins binding with SNP-D4 were separated and analyzed for mass spectrometry. Eight proteins were identified to interact characteristically with SNP-D4 in FOC4 (Table 1). Of these eight proteins, A0A5C6SPC6, which is annotated as acetaldehyde dehydrogenase, contributes to the metabolism of alcohol, oxidizing acetaldehyde and converting it into acetic acid (Chen, Cruz & Mochly-Rosen, 2015; Anand et al., 2019). Aldehydes can inhibit spore germination and have sporicidal activity, which is one of the key factors leading to spore death (Bowles & Miller, 1993). The inhibition of acetaldehyde dehydrogenase can cause the accumulation of the intermediate product acetaldehyde, which results in biological poisoning and even death (Weathermon & Crabb, 1999). So, aldehyde dehydrogenase has been considered as the target of some antibiotics (Weathermon & Crabb, 1999). In order to evaluate the credibility of the interaction, A0A5C6SPC6 was selected as an example to analyze the collaboration sites with SNP-D4 (Fig. 5), suggesting that SNP-D4 may bind to the catalytic center of A0A5C6SPC6, thereby inhibiting acetaldehyde dehydrogenase activity, and leading to the impaired metabolism of ethanol and possibly contributing to the antifungal effect. Next the spore proteins interacting with the eight proteins, which were trapped by SNP-D4, were subjected to protein-protein interaction analysis (Fig. 6, Table S1), indicating that the interference of SNP-D4 was implicated in ‘translation’, ‘folding, sorting and degradation’, ‘transcription’, ‘signal transduction’ and ‘cell growth and death’ pathways, and impairedthe growth of *F. oxysporum* broadly.

Collectively, *Fusarium oxysporum f. sp. cubense* (FOC4) is a devastating pathogen that causes rotted roots in banana and damage the banana industry. Pepaptamer SNP-D4 was identified to impede the spore germination, possibly by interfering with protein-protein interactions and blocking the target protein function in FOC4. In our future plan, a specific FOC4-resistant banana plant will be engineered that confers a high level of specificity to inhibit FOC4 by expressing SNP-D4. Another alternative biological control is to directly spray the bacterial products expressing SNP-D4 on banana plants suffering from banana wilt, so to achieve the therapeutic effect of biological pesticides. These are of positive significance for reducing the incidence of FOC4 in the banana industry and ensuring the safety of bananas for humans.

## Supplemental Information

10.7717/peerj.12756/supp-1 Supplemental Information 1 SDS-PAGE agar results of SN skeleton purification. Line 6 represents the purified products of SN protein.

10.7717/peerj.12756/supp-2 Supplemental Information 2 SDS-PAGE agar results of SNP-D4 purification. Line 6 represents the purified products.

10.7717/peerj.12756/supp-3 Supplemental Information 3 The pull down assay to isolate proteins interacting with SNP-D4. FOC4 was cultured in PDB medium, and the hyphae was filtered by gauze to collect the spores.The supernatant was incubated with SNP-D4 for 2 h and loaded on the nickel column. After the nonspecific binding proteins were eluted with 20 mM imidazole, the target proteins interacting with SNP-D4 were pulled down eluted using 80 mM imidazole.

10.7717/peerj.12756/supp-4 Supplemental Information 4 Spore proteins interacting with the eight proteins targeted by SNP-D4.

10.7717/peerj.12756/supp-5 Supplemental Information 5 Spore germination rate treated by SNP-D4. The pepaptemer concentration ranged from 0-8 u.

10.7717/peerj.12756/supp-6 Supplemental Information 6 Fungicide rate and the pepaptemer concentration ranged from 0-8 u.

## Additional Information and Declarations

### Data Availability

The following information was supplied regarding data availability: The raw data are available in the Supplemental Files.
