# Supplementary figures and images for: Antifungal activity of an artificial peptide aptamer SNP-D4 against Fusarium oxysporum
Source: PeerJ. 2022 Feb 22;10:e12756. doi: 10.7717/peerj.12756 (PMC8877334; doi:10.7717/peerj.12756)

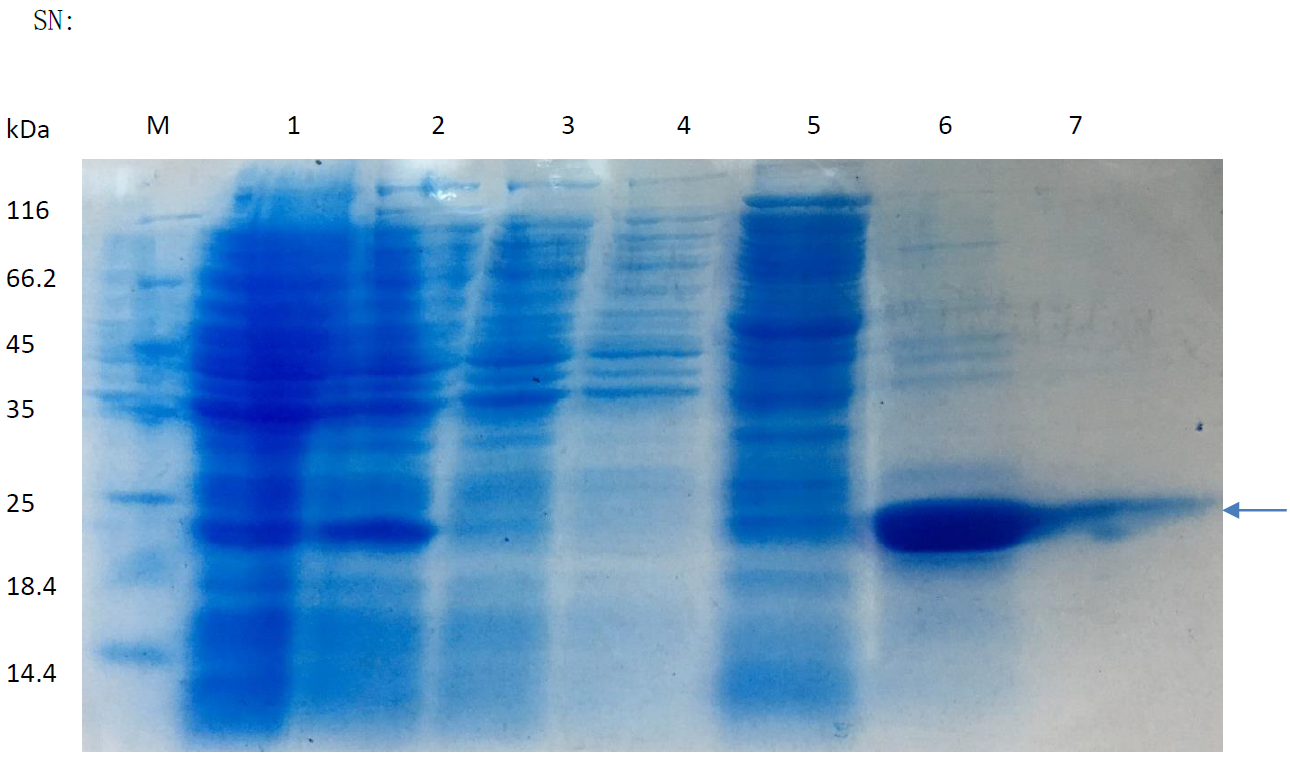

Supplement: Supplemental Information 1 — Line 6 represents the purified products of SN protein. [file peerj-10-12756-s001.png]

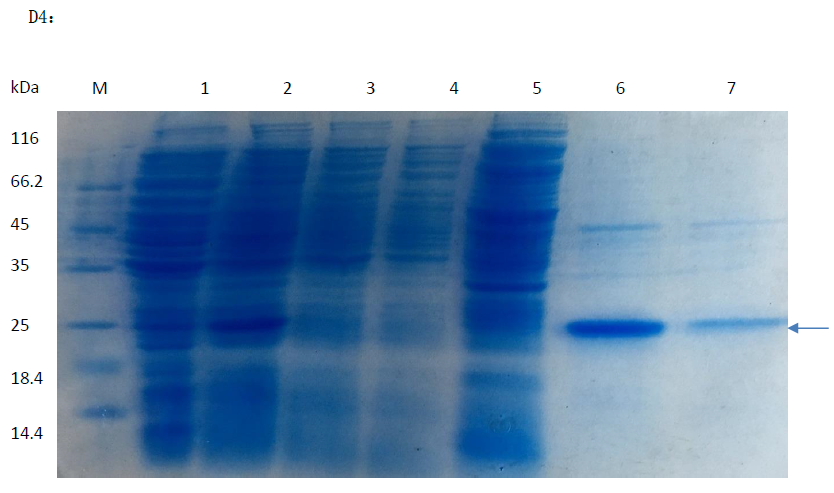

Supplement: Supplemental Information 2 — Line 6 represents the purified products. [file peerj-10-12756-s002.png]

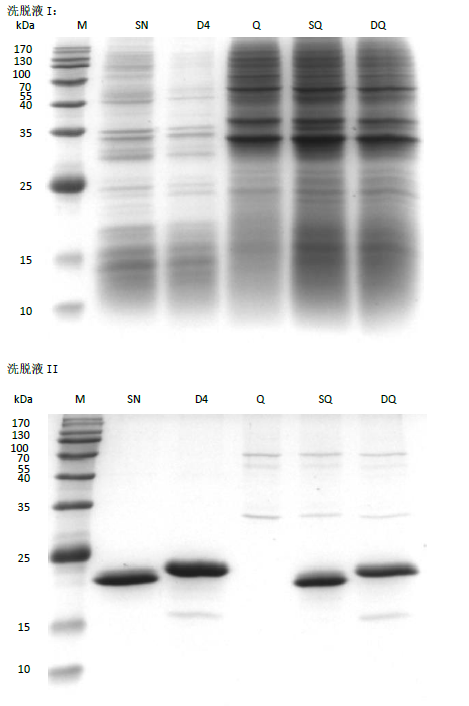

Supplement: Supplemental Information 3 — FOC4 was cultured in PDB medium, and the hyphae was filtered by gauze to collect the spores.The supernatant was incubated with SNP-D4 for 2 h and loaded on the nickel column. After the nonspecific binding proteins were eluted with 20 mM imidazole, the target proteins interacting with SNP-D4 were pulled down eluted using 80 mM imidazole. [file peerj-10-12756-s003.png]
